# Extracellular vesicles and preterm infant diseases

**DOI:** 10.3389/fped.2025.1550115

**Published:** 2025-02-17

**Authors:** Wenqain Chen, Supasek Kongsomros, Alexander Thorman, Leyla Esfandiari, Ardythe L. Morrow, Somchai Chutipongtanate, David S. Newburg

**Affiliations:** ^1^ Department of Neonatology, Fujian Maternity and Child Health Hospital; College of Clinical Medicine for Obstetrics & Gynecology and Pediatrics, Fujian Medical University, Fuzhou, Fujian, China; ^2^ MILCH and Novel Therapeutics Lab, Division of Epidemiology, Department of Environmental and Public Health Sciences, University of Cincinnati College of Medicine, Cincinnati, OH, United States; ^3^ Department of Biomedical Engineering, University of Cincinnati College of Engineering, Cincinnati, OH, United States; ^4^ Extracellular Vesicle Working Group, University of Cincinnati College of Medicine, Cincinnati, OH, United States; ^5^ Department of Infectious Disease, Cincinnati Children’s Hospital Medical Center, Cincinnati, OH, United States

**Keywords:** preterm infants, extracellular vesicles, diseases, biomarkers, therapeutics

## Abstract

With the continuous improvement in perinatal care, the number of viable preterm infants is gradually increasing, along with the rise in preterm-related diseases such as necrotizing enterocolitis, bronchopulmonary dysplasia, perinatal brain injury, retinopathy of prematurity, and sepsis. Due to the unique pathophysiology of preterm infants, diagnosing and treating these diseases has become particularly challenging, significantly affecting their survival rate and long-term quality of life. Extracellular vesicles (EVs), as key mediators of intercellular communication, play an important regulatory role in the pathophysiology of these diseases. Because of their biological characteristics, EVs could serve as biomarkers and potential therapeutic agents for preterm-related diseases. This review summarizes the biological properties of EVs, their relationship with preterm-related diseases, and their prospects for diagnosis and treatment. EVs face unique challenges and opportunities for clinical applications.

## 1 Introduction

Globally, approximately 15 million premature births occur annually, representing an estimated 11% of all deliveries (1). Advances in perinatal care and neonatal resuscitation techniques have increased the prevalence of preterm births, leading to a rise in associated complications among preterm infants, including necrotizing enterocolitis (NEC), bronchopulmonary dysplasia (BPD), perinatal brain injury (PBI), retinopathy of prematurity (ROP), and sepsis (2–5). Over the past decade, these complications have remained a significant cause of neonatal mortality and emerged as a leading cause of death among children under five years old (6). These complications not only profoundly impact the survival rate and long-term quality of life of preterm infants, but also impose psychological stress on families and incur substantial economic costs (7).

Early detection and treatment of these diseases have become urgent priorities to reduce the incidence of preterm birth and enhance the survival quality and long-term prognosis of preterm infants. As these diseases occur in immature and developing organs and involve complex underlying pathophysiological mechanisms, reliable diagnostic tools and therapeutic interventions are currently lacking for many of them.

**Figure 1:**
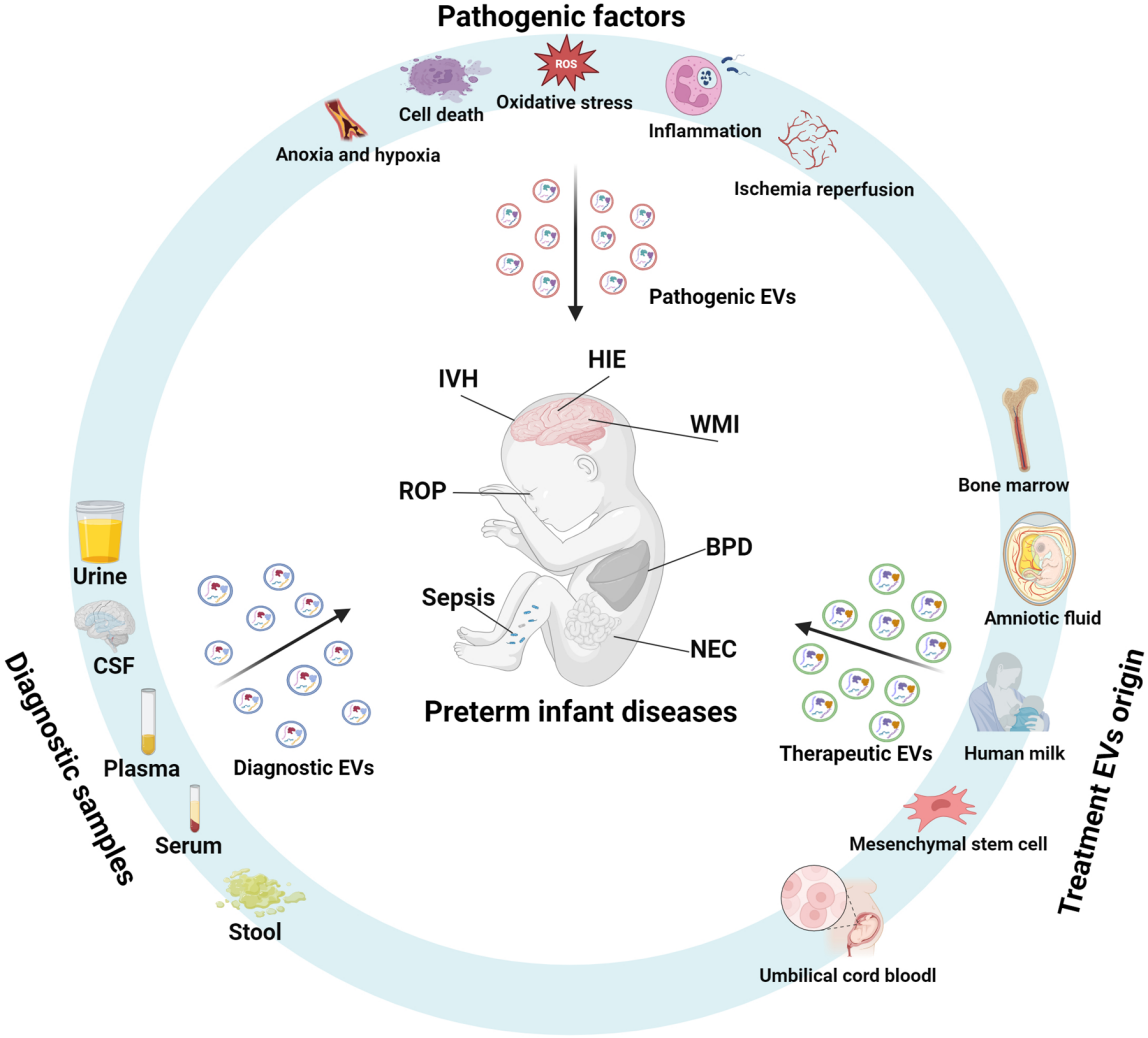
Extracellular vesicles in the pathophysiology, diagnosis, and therapy of preterm-related diseases. During pathological processes such as inflammation, ischemia-hypoxia, cell death, oxidative stress, and ischemia-reperfusion, extracellular vesicles (EVs) act as mediators of intercellular signaling, playing a role in the pathophysiology of preterm-related diseases. These diseases affect the quantity and composition of EV cargo, and analyzing the EVs in body fluids or tissue samples can aid in disease diagnosis. EVs derived from mesenchymal stem cells, breast milk, amniotic fluid, umbilical cord blood, and bone marrow exhibit anti-inflammatory, antioxidant, and cell regeneration-promoting properties, making them promising therapeutic agents for preterm-related diseases. (Created with BioRender.com).

Extracellular vesicles (EVs) are mediators of intercellular signaling and play regulatory roles in the pathophysiological processes of preterm-related diseases. The lipid bilayer of EVs protects their cargoes from degradation, giving EVs unique characteristics that hold promise as biomarkers for diagnosing preterm infant-related diseases and as therapeutic tools (Figure 1). This review summarizes the biological characteristics of EVs and their relationship with preterm delivery, focuses on the role of EVs in complications associated with preterm infants, and discusses their potential as diagnostic and therapeutic tools. Finally, it highlights key issues that need to be addressed to allow the clinical application of EVs.

## 2 The biological characteristics of extracellular vesicles

Extracellular vesicles (EVs) are non-replicating, lipid bilayer-bound vesicles released from cells into the extracellular space (8). They are classified into exosomes, microvesicles, and apoptotic bodies based on their biogenesis, cellular origin, and biophysical properties (9). Exosomes, typically 40–150 nm in diameter, originate from the inward budding of the endosomal membrane to form intraluminal vesicles within multivesicular bodies. These intraluminal vesicles are released into the extracellular space as exosomes upon the fusion of multivesicular bodies with the plasma membrane (10, 11). The exosome biogenesis is regulated by the endosomal sorting complex required for transport (ESCRT)-dependent or ESCRT-independent pathways (12), involving specific sorting and packaging of cargo into exosomes (13). Microvesicles, approximately 100–1,000 nm in diameter, bud directly from the plasma membrane, enclosing cytoplasmic contents, and are typically released during cellular stress and activation (10, 11). Apoptotic bodies, with diameters ranging from 500–2000 nm, form during cell apoptosis, characterized by membrane shrinkage and invagination, leading to the packaging of cytoplasmic material, including DNA and organelles (14).

EVs carry diverse cargoes, which vary by cell type and cellular status, affecting their function and fate (15, 16). EVs have pivotal roles in physiology (17), immunology (18), and metabolism (19). EVs function as cell-to-cell messengers by transferring mRNA that, upon entering cells, are translated into specific proteins with unique biological effects (20). Besides mRNA, EVs transport various molecules between cells, including proteins, lipids, DNA, and non-coding RNA, making them vital regulators of cellular communication (20, 21).They are detectable in body fluids such as blood, saliva, and urine, thereby offering a convenient means for disease detection (22). As the cargo of EVs is cell-specific, reflecting their cells of origin (15, 16), EVs can be used as biomarkers for studying specific cell types involved in various diseases. EV concentration can indicate disease progression, with studies achieving high accuracy in distinguishing the severity of bronchopulmonary dysplasia (BPD) based on EV levels (23).

EVs also hold therapeutic promise (24). They carry molecules capable of modifying cell signaling and gene expression, thereby exerting therapeutic effects (25, 26). Compared to traditional drug delivery methods, EVs offer advantages such as enhanced cargo protection and tissue penetration (27). Derived from benign sources, therapeutic EVs are less likely to provoke adverse reactions, which can be further improved by reducing surface proteins (28, 29). Furthermore, they can be engineered for targeted delivery, thereby enhancing their efficacy (30).

As interest in EVs as potential biomarkers and therapeutics grows and EV research has significantly increased, the International Society for Extracellular Vesicles introduced the Minimal information for studies of extracellular vesicles guidelines to standardize protocols and reporting (8, 31). These guidelines cover nomenclature, separation techniques, characterization, functional studies, and sample collection. However, they discourage using exosomes or microvesicles unless their subcellular origin is confirmed but recommend using EV with terms based on size, density, molecular composition, or cellular origin (8).

## 3 Role of extracellular vesicles in preterm infant diseases

From a clinical viewpoint, there are three major roles of EVs in preterm infant diseases: pathogenic EVs, diagnostic EVs, and therapeutic EVs. Pathogenic EVs typically originate from damaged cells or diseased tissues and are enriched with pro-inflammatory factors and damage-related molecules, directly contributing to disease progression (32, 33). Diagnostic EVs, derived from body fluids, can be obtained non-invasively and carry disease-specific biomarkers, making them suitable for early diagnosis and real-time monitoring (34, 35). Therapeutic EVs generally come from stem cells or plant/animal extracts and are modified to enhance their drug or gene delivery capabilities, intervening in pathological processes and promoting tissue repair (36, 37). In terms of composition and function, pathogenic EVs carry inflammatory factors and pathological mediators that drive disease progression, while diagnostic EVs contain highly specific biomarkers that aid in disease detection (38, 39). Therapeutic EVs deliver drugs, RNA, or targeted molecules for therapeutic intervention (36). While these three types of EVs differ in their origin, composition and function, they all demonstrate significant clinical translational potential and complement each other, advancing the application of extracellular vesicles in neonatal disease research and therapy.

## 4 Extracellular vesicles and spontaneous preterm birth

Childbirth represents a complex interplay between the fetus and the mother, where factors such as fetal endocrine signals, maternal endocrine signals, other signaling, and immune changes play crucial roles in maintaining pregnancy (40, 41). Disruptions in the balance of endocrine and immune systems can lead to an overload of inflammation, ultimately culminating in spontaneous preterm birth (42). This process shares similarities with full-term delivery, involving heightened uterine contractions, cervical dilation, and rupture of fetal membranes, all triggered by a transition in the uterine muscle layer from a quiescent state to intermittent contractions (43). Progesterone plays a key role in inhibiting the expression of pro-inflammatory factors to maintain the quiescent state of the uterine muscle layer (44). Fetal inflammatory signals can lead to functional progesterone withdrawal, increased intrauterine inflammatory factors, immune cell activation, disruption of maternal inflammatory balance, and ultimately preterm delivery (42).

EVs are significant players in the pathophysiological processes of spontaneous preterm birth. In a mouse model, EVs carrying inflammatory mediators increase gradually from day 5–19 of pregnancy. Late pregnancy EVs, when injected into mice at day 15 of pregnancy, induce preterm birth and related inflammation (45). This suggests that EVs regulate parturition through paracrine signaling. Menon et al. (46) discovered decreased placental-derived EVs in maternal plasma of preterm birth compared to term birth mothers, with significant differences in protein composition associated with inflammation, epithelial-mesenchymal transition, coagulation/complement activation, and cell death. Another study compared content in maternal plasma between different preterm birth causes, revealing variations in total circulating EV protein mainly related to inflammation and metabolic signaling (47). Gray et al. (48) observed dysregulation of circulating miRNAs in plasma of spontaneous preterm birth compared to normal pregnancies. Analysis of EV miRNA characteristics between term and preterm deliveries identified differences in miRNAs targeting signaling pathways such as TGF-β, p53, and glucocorticoid receptor signaling, implicating circulating EV miRNAs in preterm birth mechanisms (49). McElrath et al. (50) explored the potential of EVs isolated from maternal plasma in the first trimester of singleton pregnancies as biomarkers for spontaneous preterm birth before 35 weeks, identifying 5 EV proteins as predictive markers with promising diagnostic performance. Zhao et al. (51) analyzed EV lipids in maternal plasma during mid-pregnancy, identifying microvesicle phosphatidyl serine (34:0) as a potential predictor for preterm birth.

EVs also hold therapeutic potential in spontaneous premature birth research. Sheller-Miller et al. (52) designed EVs containing NF-*κ*B inhibitors, demonstrating their ability to prolong gestation and reduce maternal inflammation, suggesting EVs could serve as stable and specific interventions to mitigate inflammation associated with preterm birth.

## 5 Extracellular vesicles and necrotizing enterocolitis

Necrotizing enterocolitis (NEC) poses a significant threat to preterm infants, representing a common and often life-threatening gastrointestinal emergency. Global incidence rates have seen a troubling increase over the past decade, particularly among premature infants weighing less than 1,000 grams at birth (53). Due to the challenges in early diagnosis and the lack of effective treatments, NEC often progresses rapidly, with mortality rates estimated around 25% and reaching up to 80% in severe cases of fulminant NEC (54).

The pathogenesis of NEC is multifaceted, closely intertwined with intestinal epithelial damage, mucosal repair mechanisms, and inflammatory responses; each can be regulated by EVs. EVs derived from intestinal epithelial cells activate wound repair pathways (55) and contribute to maintaining intestinal immune balance (38). Post-injury, intestinal epithelial cells release EVs into the mesenteric lymph, and these EVs have immunomodulatory effects that suppress post-injury inflammatory signaling and NEC progression (33, 38). Additionally, polymorphonuclear neutrophils release EVs during NEC, triggering acute remodeling of epithelial junctions, enhancing neutrophil recruitment, and exacerbating epithelial damage (56). Moreover, adherent-invasive *Escherichia coli* (AIEC) infection can boost EV secretion from intestinal epithelial cells, with these EVs promoting AIEC replication and inducing pro-inflammatory responses (57). This evidence underscores the influence of EVs on NEC occurrence and development through intercellular communication.

Early diagnosis of NEC is paramount for reducing morbidity and mortality rates, yet reliable biomarkers for early diagnosis remain elusive (58). EVs have potential for biomarkers for early NEC diagnosis (Table 1). Significant changes in urinary EV-derived miRNA (including miR-376a, miR-518a-3p, and miR-604) in NEC cases relative to non-NEC sepsis and healthy controls suggest urinary EV-miRNA as potential specific biomarkers for NEC (35).

**Table 1:** Application of extracellular vesicles in the diagnosis of preterm infant diseases.

Condition	Extracellular vesicle markers	Study design	Key findings	Reference
NEC	Urine derived EV miRNAs	Case-control	Multiple miRNAs including miR-376a, miR-518a-3p, and miR-604 can distinguish between NEC and non-NEC	PMID: 33785202
BPD	The quantity of EVs from tracheal aspirates and the level of miR-876-3p in EVs	Cohort, *in vivo*	Severe BPD had more EVs and lower levels of miR-876-3p	PMID: 29515035
	Surface proteins derived from tracheal aspirates EV	Cohort	Increased CD24 and CD14 on EV surface can predict BPD	PMID: 36719083
	Serum EV miR-21	Case-control, *in vivo*	miR-21 in serum EV increased at 28 days in BPD preterm infants	PMID: 32191117
HIE	Serum EVs derived from CNS	Cohort	Increasing synaptopodin and decreasing lipocalin-2 in EV had negative predictive values of 70.0% and 90.9% for HIE respectively	PMID: 34021027
IVH	MiRNAs in CSF EVs (miR-9, miR-17, miR-26a, miR-124, miR-1911)	Case-control	MiRNAs in EVs from CSF can predict post-hemorrhagic hydrocephalus in IVH patients	PMID: 30639393
WMI	miR-9 in fetal CNS-EV from maternal plasma	Case-control	Fetal CNS-EV from maternal plasma can evaluate abnormal proliferation and differentiation of fetal CNS stem cells	PMID: 31069822
	Neuronal EVs purified from peripheral blood samples	Cohort	Neuronal EV synaptopodin from peripheral blood can be a marker of brain injury	PMID: 29376087
Sepsis	The number of EVs from plasma	Case-control	The amount of EVs in plasma was positively correlated with sepsis severity	PMID: 31632618
	The number of EVs from plasma	Cohort	Elevated plasma exosome levels were associated with organ failure severity and predictive of mortality in sepsis patients	PMID: 32639098
	miRNA in serum EV	Cohort	miR-1246, miR-542-3p, and miR-193a-5p levels in plasma EVs were associated with sepsis risk and severity	PMID: 32916773
	CircRNA in serum EV	Case-control	hsa_circRNA_104484 and hsa_circRNA_104670 from serum EV could be novel diagnostic biomarkers	PMID: 34238972
	miR-34a, miR-15a, and miR-27a from endothelial progenitor cells EV	Case-control	Elevated miR-34a and decreased miR-15a and miR-27a in EVs predict septic shock occurrence	PMID: 26683209

EVs also offer promise as a novel therapeutic approach for NEC (Table 2). EVs derived from bone marrow mesenchymal stem cells (MSC) have shown potential in restoring intestinal barrier function, akin to bone marrow mesenchymal stem cells infusion alone, indicating their potential as cell-free therapy for neonatal NEC (59). MSC-EVs containing miRNAs specific to Snail/Claudin signaling pathways have induced improvements in intestinal barrier function (36). EVs from other stem cell sources, such as amniotic fluid-derived MSC, amniotic fluid-derived neural stem cells, bone marrow-derived MSC, and neonatal intestinal neural stem cells, have similarly exhibited therapeutic effects in reducing experimentally induced NEC incidence (60).

**Table 2:** Application of extracellular vesicles in the treatment of preterm infant diseases.

Condition	Extracellular vesicle source	Study design	Key findings	Reference
NEC	BM-MSCs	*in vitro*, *in vivo*	Alleviating tissue damage and protecting intestinal barrier function	PMID: 7015901
	BM-MSCs	Case-control, *in vivo*	Improvement of I/R-induced intestinal damage via the Snail/Claudins signaling pathway	PMID: 2603821
	BM-MSCs, AF-MSCs, AF-NSCs, E-NSCs	*in vivo*	Reduce the incidence of NEC	PMID: 9661576
	Human milk	*in vitro*	Support epithelial barrier function by facilitating cell migration via the p38 MAPK pathway	PMID: 33732416
	Human milk	*in vitro*	Inhibit intestinal epithelial cell death	PMID: 9991305
	Human milk	*in vitro*	Protect intestinal stem cells from oxidative stress	PMID: 2193954
	Human milk	*in vitro*, *in vivo*	Decreased inflammation and NEC-induced mucosal injury	PMID: 1713717
	Human milk	*in vivo*	Inhibit inflammation and improve intercellular tight junctions by miR-148a-3p/p53/SIRT1 axis	PMID: 5091894
	Human milk	*in vivo*	lncRNA and miRNA in EVs reduce disease severity and promote intestinal cell proliferation	PMID: 36448375
	Human milk	*in vitro*, *in vivo*	Induces epithelial regeneration, reduces inflammation and fibrosis, and regulates immune response	PMID: 38054009
BPD	MSCs	*in vivo*	MSC-EVs improved lung function and vascularization and reduced inflammation in BPD animal models	PMID: 33502939, PMID: 28853608
	Human umbilical cord MSC	*in vivo*	Alleviate lung injury in BPD rat model by affecting cell survival and angiogenesis	PMID: 33040709
	BM-MSCs	*in vitro*	miR-425 in EVs inhibits hyperoxia-induced lung injury by targeting PTEN and upregulating the PI3 K/AKT axis	PMID: 33264631
	Adipose MSCs	*in vitro*, *in vivo*	Adipose MSC-EVs carrying miR-21-5p alleviated hyperoxia-induced lung injury via the SKP2/Nr2f2/C/EBP*α* axis	PMID: 34882302
	BM-MSCs	*in vivo*	BM-SC-EVs miR-34c-5p reduced lung injury and inflammation in BPD by blocking the OTUD3/PTEN axis	PMID: 37310728
	Human milk	*in vitro*	HMEV inhibit type II alveolar epithelium cell apoptosis to prevent BPD	PMID: 35833257
	Human milk	*in vitro*, *in vivo*	HMEV-circDNAJB6 reduced damage and suppressed the proliferation of alveolar epithelial cells in the BPD model	PMID: 38244155
	Human milk	*in vitro*	HMEV-circABPD1 protected against BPD by promoting cell proliferation, reducing oxidative stress, and alleviating lung injury via the miR-330-3p/HIF1α axis	PMID: 37660980
HIE	MSCs	*in vivo*	MSC-EVs can improve brain functional impairment, reduce seizure frequency and duration, and restore subcortical white matter myelination	PMID: 3991170
	MSCs	*in vivo*	MSC-EVs comparably protected neonatal mice from HIE-induced brain tissue atrophy	PMID: 7069694
	MSCs	*in vivo*	EV modulation of the PI3 K/AKT signaling pathway to inhibit calcium overload and neuronal cell death	PMID: 36147480
	MSCs	*in vivo*	MSC-EVs have a neuroprotective effect by preventing HIE-induced blood-brain barrier leakage via Annexin A1	PMID: 30682787
	BM-MSCs	*in vivo*	BM-MSCs can regulate the polarization and inflammatory response of microglia in HIE patients	PMID: 35259691
	Astrocyte	*in vivo*	Astrocyte-EVs inhibited hippocampal immune cells by delivering miR-124-3p in HIE mice	PMID: 37748110
	BM-MSCs	*in vivo*	miR-410 from BM-MSC EVs inhibits neuronal apoptosis induced by HIE	PMID: 29562785
	MSCs	*in vivo*	miR-21a-5p from MSC-EVs exert anti-inflammatory and anti-apoptotic effects	PMID: 32619670
	Wharton's jelly MSCs	*in vitro*	EV-derived miR-let-7-5p prevent and resolve HIE-induced apoptosis	PMID: 29562785, PMID: 32858071
	Astrocyte	*in vivo*	Astrocyte-derived EVs containing miR-17-5p alleviate neuronal apoptosis and inflammation in HIE neonatal rats	PMID: 33309839
	BM-MSCs	*in vitro*	miR-93 delivered by BM-MSC-EV alleviates neuronal apoptosis and inflammation in HIE mice through JMJD3-dependent p53/KLF2 axis	PMID: 35952773
	NSCs	*in vitro*	miR-150-3p in NSC-EV inhibits neuronal apoptosis and promotes proliferation after HIE by targeting CASP2	PMID: 35436510
	NSCs	*in vitro*	NSC-EV promote neuronal survival, inhibit apoptosis, enhance Nrf2 nuclear translocation to counter oxidative stress, and foster axonal growth and angiogenesis	PMID: 32437794
IVH	MSCs	*in vivo*, *in vitro*	MSC-EV attenuated neuronal cell death and severe IVH-induced brain injury via brain-derived neurotrophic factor	PMID: 33319929
	BM-MSCs	*in vivo*	miR-146a-5p-enriched BM-MSC-EVs protect neurons and improve function after IVH by reducing apoptosis, inflammation, and microglial M1 polarization	PMID: 32821084
WMI	Wharton's jelly-MSCs	*in vitro*, *in vivo*	EVs from Wharton's jelly-MSC have anti-inflammatory effects on microglia-mediated neuroinflammation in perinatal brain injury	PMID: 30898154
	MSCs	*in vivo*	MSC-EVs ameliorate inflammation-induced cellular damage in a rat model of preterm brain injury	PMID: 27847282
	Wharton's jelly-MSCs	*in vivo*	MSC-EVs rescued normal myelination, mature oligodendroglial, and neuronal cell counts, which were impaired after perinatal brain injury	PMID: 31398924
PAIS	MSCs	*in vivo*	MSC-EVs accumulate in the ipsilateral hemisphere of occluded neonatal stroke, preventing perinatal arterial ischemic stroke through interactions with microglia	PMID: 34235636
ROP	BM-MSCs	*in vivo*	Intravitreal administration of MSC-EVs reduced the severity of ROP	PMID: 28636406
	MSCs	*in vitro*, *in vivo*	MSC-EVs alleviate neuroinflammation and cell apoptosis induced by ROP injury	PMID: 30654160
	Microglial cells	*in vivo*	miR-24-3p derived from microglia EV can reduce photoreceptor damage of ROP and promote normal blood vessel formation	PMID: 31163320
	Lymphocytic microparticles	*in vitro*, *in vivo*	miR-181a in lymphocyte microparticles can inhibit ROP retinal angiogenesis	PMID: 31163320, PMID: 29608244
Sepsis	BM-MSCs	*in vitro*	miR-17 derived from BM-MSC-EVs regulates BRD4-mediated EZH2/TRAIL axis to inhibit inflammation	PMID: 33915488
	Adipose tissue, bone marrow, and umbilical cord MSCs	*in vitro*, *in vivo*	MSC-EVs alleviate systemic inflammatory response and protect lung tissues in sepsis	PMID: 35265265
	Adipose MSCs	*in vitro*, *in vivo*	Adipose MSC-EV inhibited IL-27 secretion in macrophages and alleviated sepsis-induced acute lung injury	PMID: 35013123

Human milk-derived EVs (HMEVs) have potential to prevent NEC in premature infants (37). HMEVs contribute to intestinal development, maintain barrier function, and offer protective effects against NEC. HMEVs have been shown to enhance cell migration, protect against oxidative stress-induced damage, and promote intestinal stem cell survival through various signaling pathways, thereby preventing and treating NEC (61–63). Additionally, HMEVs contain miRNAs and other bioactive molecules that regulate immune responses and inflammation, further mitigating NEC severity (64, 65). Proteomic analysis has identified lactoferrin as a key cargo of HMEVs with protective properties against NEC (66). Furthermore, the omega-3 fatty acids present in HMEVs contribute to intestinal epithelial reformation, fibrosis alleviation, and immune response regulation (67).

## 6 Extracellular vesicles and bronchopulmonary dysplasia

Bronchopulmonary dysplasia (BPD) is a multifactorial chronic lung disease commonly associated with prematurity and a leading cause of respiratory disease-related mortality in premature infants (68). The global incidence of BPD is estimated to range from 11% to 50% (69). With advances in perinatal medicine, the survival rate of extremely premature infants has notably increased, with a parallel increase in BPD incidence (70–72). Long-term complications that follow preterm BPD, such as neurodevelopmental impairment and cardiopulmonary dysfunction, result in a significant social burden (73).

BPD is associated with abnormal prenatal repair and repetitive postnatal lung injuries, characterized by pulmonary airway and vascular system inflammation and destruction, leading to alveolar simplification, pulmonary fibrosis, and pulmonary hypertension (69). EVs are implicated in these pathological processes. Genschmer et al. (32) demonstrated that EVs derived from infants with severe BPD could induce lung parenchymal simplification, increased airway resistance, and right ventricular hypertrophy in newborn mice, whereas those from non-BPD infants did not cause lung injury. Premature infants, due to incomplete lung development and inadequate surfactant production, often require high-concentration oxygen therapy and mechanical ventilation, both of which contribute to lung injury (74). EVs isolated from rats exposed to high oxygen levels exacerbate lung injury associated with BPD, and concentrations of EV particles are elevated in tracheal aspirates of infants with severe BPD, suggesting a role of EVs in BPD pathogenesis (75).

EVs carry specific proteins or RNA molecules relevant to lung diseases and can serve as biomarkers for predicting BPD (Table 1). Lal et al. (23) identified EV-derived miR876-3p as a potential biomarker for severe BPD in premature infants, with reduced expression at birth predicting future development of severe BPD. Likewise, increases in EV specific surface proteins (CD24 and CD14) during lung development are associated with elevated BPD risk (76). Serum EV-miRNA-21 was upregulated in premature infants with BPD, suggesting its potential as an early biomarker for BPD development (34).

Considerable research has explored the use of EVs in BPD treatment (Table 2). Among various delivery methods, intratracheal administration is considered the preferred approach for treating severe lung diseases due to its ability to directly target the affected area, provide high local drug delivery, and minimize systemic toxicity. Additionally, it offers a needle-free route with rapid onset, low metabolism, and high bioavailability (77). This method is already widely used in clinical treatments for lung diseases in preterm infants (78). Intratracheal administration of MSC-EVs improves lung function, promotes vascularization, and reduces inflammation in BPD animal models (79, 80). This therapeutic effect is associated with changes in molecular pathways, such as PTEN/Akt and miRNAs, involved in BPD pathogenesis (81–84).

Moreover, human milk feeding has been linked to a reduced incidence of BPD in infants, with HMEVs playing a protective role in lung epithelial cells in rats (85–87). Circulating RNA molecules, such as circDNAJB6 and circABPD1 derived from HMEVs, have shown potential in alleviating BPD pathology (88, 89).

## 7 Extracellular vesicles and perinatal brain injury

Premature infants face a heightened risk of perinatal brain injury, with the likelihood of long-term neurological impairment increasing as gestational age decreases, reaching a lifetime disability rate of up to 5.2% among extremely premature infants (90). The pathophysiological mechanisms underlying brain injury in premature infants are multifaceted, involving prenatal factors such as intrauterine infections and chorioamnionitis, perinatal factors such as birth asphyxia, and postnatal factors including hemorrhage, infection, and mechanical ventilation. EVs serving as intercellular messengers significantly influence the pathophysiology, diagnosis, and treatment of perinatal brain injury in premature infants (Tables 1, 2). The roles of EVs in several types of perinatal brain injury are summarized as follows.

### 7.1 Hypoxic-ischemic encephalopathy

Hypoxic-ischemic encephalopathy (HIE) in newborns is a primary cause of perinatal brain injury, arising from hypoxia-ischemia during the perinatal period, culminating in devastating consequences. In developed nations, HIE's incidence is estimated at 1–6 cases per 1,000 live births, constituting 15%–35% of all neonatal brain disorders (91, 92), with a mortality rate accounting for 23% of global neonatal deaths (93). Therapeutic hypothermia is currently the most effective method for treating HIE. However, even with hypothermia therapy, approximately 30% of survivors endure long-term severe neurodevelopmental disorders, including sensory, cognitive, and neuropsychological deficits (94, 95).

Throughout the pathophysiological cascade of HIE, involving ischemia-hypoxia and subsequent ischemia-reperfusion, neuronal cell damage particularly affects oligodendrocytes. Neuronal EVs likely exert regulatory roles in HIE pathogenesis. Chiang et al. (96) observed significant differences in expression levels of 45 EV-derived miRNAs between normoxic and ischemic/reperfused neuronal models. Functional analysis of these differentially expressed EV-miRNAs implicated their involvement in various pathways related to cell survival and death, neuronal signaling, and dendritic growth, underscoring a pivotal role of EVs in HIE pathogenesis (96).

Research on the use of EVs as diagnostic biomarkers for neonatal HIE is limited. Pineles et al. (39) purified central nervous system-derived EVs from serum of term and near-term infants treated with hypothermia. The protein levels of EVs at different time points significantly correlated with the severity of HIE, with decreased levels of synaptic proteins between 0 and 12 h after birth and increased levels of lipocalin-2 between 12 and 48 h after birth (39). The negative predictive values for increased synaptic proteins was 70% and decreased lipocalin-2 was 91%, suggesting that the content of central nervous system EVs in peripheral blood can serve as a biomarker for the severity of HIE and response to hypothermia therapy (39).

Currently, the only proven effective therapy for HIE is therapeutic hypothermia, but due to the short treatment window (within 6 h after birth) and unsuitability for premature infants with gestational age <35 weeks (97), researchers are exploring the combined use of EVs to improve HIE treatment and outcomes. In an HIE sheep model, human MSC-EVs ameliorated brain function impairment, reduced seizure frequency and duration, and restored subcortical white matter myelination (98). Intranasally administered EVs derived from immortalized mesenchymal stromal cells mitigate neuronal damage in neonatal HIE by suppressing neuroinflammation and fostering neuroregeneration, thereby attenuating long-term cognitive deficits and behavioral abnormalities (99, 100). These protective effects are mediated through EV modulation of the PI3K/AKT signaling pathway, which inhibits calcium overload and neuronal cell death (101), prevention of HIE-induced blood-brain barrier leakage via targeting the membrane-associated protein A1/formylpeptide receptor axis (102), and immunomodulation (103). Additionally, miRNAs encapsulated within EVs are potent mediators of neuroprotection against HIE-induced neuronal damage. EVs derived from astrocytes deliver miR-124-3p to inhibit abnormal activation of hippocampal immune cells in HIE (104). MiR-410 from bone marrow MSC EVs inhibits neuronal apoptosis induced by HIE (105). EVs containing miR-21a-5p exert anti-inflammatory and anti-apoptotic effects (106). Human MSC-EV cargo miR-let-7-5p has neuroprotective and anti-inflammatory effects; pretreatment with hydrogen sulfide enhances their neuroprotective capabilities (105, 107). Astrocyte-derived EVs containing miR-17-5p alleviate neuronal apoptosis and inflammation in HIE neonatal rats (108). Additionally, miR-93 in MSC-EVs inhibits HIE-induced neuronal damage through the JMJD3-dependent p53/KLF2 signaling axis, while miR-150-3p in neural stem cell-derived EVs protects the central nervous system from ischemia-reperfusion injury (109, 110). EVs derived from neural stem cells promote neuronal survival, inhibit apoptosis, enhance Nrf2 nuclear translocation to counter oxidative stress, and foster axonal growth and angiogenesis (111).

### 7.2 Intraventricular hemorrhage

Intraventricular Hemorrhage (IVH) is one of the most common neurological complications in premature infants, occurring in an estimated 25%–30% of VLBW infants (112). The pathophysiology of IVH is related to the inherent fragility of the germinal matrix in premature infants and disruption of cerebral blood flow (113). Increased severity of intraventricular hemorrhage (IVH) increases risk of adverse neurodevelopmental outcomes. The most common complications after IVH are post-hemorrhagic hydrocephalus (PHH) and periventricular leukomalacia (PVL). Analysis of EVs from cerebrospinal fluid (CSF) in patients with PHH found enrichment of miRNAs such as miR-9, miR-17, miR-26a, miR-124, and miR-1911, suggesting that miRNAs in EVs from CSF of IVH patients could be used as biomarkers for predicting PHH (114). Studies have found that EVs also have neuroprotective effects against IVH. Brain-derived neurotrophic factor in MSC-EVs can mitigate IVH-induced neuroinflammation and cell apoptosis, and prevent the progression of post-hemorrhagic hydrocephalus, improving prognosis (115). After intracerebral hemorrhage, MiR-146a-5p in MSC-EVs can inhibit neuronal apoptosis and provide neuroprotection and functional improvement by suppressing the expression of IRAK1 and NFAT5, thus inhibiting inflammation associated with M1 polarization of microglia (116).

### 7.3 White matter injury

Various perinatal insults culminate in focal cystic necrosis and/or diffuse white matter injury (WMI) in the central nervous system, with astrocyte hypertrophy (gliosis), microglial activation, decreased white matter volume, and impaired myelination (117). The incidence of WMI in premature infants is 33%, which increases with decreasing gestational age (118). WMI correlates with adverse cognitive, language, and behavioral outcomes in premature infants (119) and is a major contributor to cerebral palsy (120).

The etiology of WMI in premature infants is multifactorial, with inflammation playing a pivotal role in its pathogenesis (121). Systemic inflammation activates microglia and astrocytes with production of pro-inflammatory mediators disrupting the blood-brain barrier, allowing systemic proinflammatory molecules to further exacerbate brain injury (122). During WMI, EVs from astrocytes enter the peripheral circulation (123), promoting leukocyte migration into the brain by inhibiting peroxisome proliferator-activated receptor α, thereby inducing inflammation in brain. EVs from microglia containing abundant TNF-α can induce reactive astrocyte transformation and demyelination (124).

Fetal central nervous system EVs can traverse the blood-brain barrier and placental barrier to enter the maternal circulation, rendering them potential early biomarkers for perinatal brain injury (125). After ethanol exposure in early pregnancy, such EVs derived from the fetal central nervous system and isolated from maternal plasma predicted adverse fetal neurological outcomes (126). Similarly, a biomarker for acute brain injury can be levels of synaptotagmin in neuron-derived EVs purified from peripheral blood samples (127).

Current treatments for WMI primarily focus on promoting neural recovery, but EVs exhibit neuroprotective effects against WMI. In response to lipopolysaccharide activation of microglia *in vitro*, MSC-EVs reduce the production of pro-inflammatory cytokines (128). In an animal model of inflammation-induced WMI, MSC-EVs reduced inflammation-induced neuronal cell degeneration, reduced microglial proliferation, and prevented reactive astrocyte proliferation (129). MSC-EV administration restored short-term myelination defects and long-term microstructural abnormalities in white matter, thereby improving persistent cognitive function (129). In a model of hypoxia combined with inflammation-induced white matter injury, MSC-EVs promoted normal myelination of damaged neurons, facilitated oligodendrocyte maturation, and supported regeneration of neuronal cells, significantly enhancing learning ability in animals with WMI (130).

### 7.4 Perinatal arterial ischemic stroke

Perinatal arterial ischemic stroke, with an incidence of approximately 1 in 2,300, is associated with severe long-term neurological and cognitive deficits, including cerebral palsy and developmental disorders (131). Arterial ischemic stroke is an occlusive cerebrovascular event, usually thrombotic in nature, with an unclear pathogenesis. One study reported that MSC-EVs administered via intraventricular or intranasal routes accumulate in the ipsilateral hemisphere of occluded neonatal stroke, preventing perinatal arterial ischemic stroke through interactions with microglia (132).

## 8 Extracellular vesicles and retinopathy of prematurity

Retinopathy of Prematurity (ROP), a potentially blinding vascular proliferative retinal disease, is the second leading cause of blindness in children in the United States (133). Two main factors contributing to the pathogenesis of ROP are immaturity of retinal vasculature and oxidative damage caused by hyperbaric oxygen exposure (134). Prematurity can include retinal vascular immaturity, making it susceptible to retinal damage when exposed to high oxygen levels, sometimes even in ambient air. Hypoxia-inducible factor 1*α* is reduced by elevated oxygen levels, reducing levels of VEGF and IGF-1, thereby inhibiting retinal vascular growth. Impaired retinal vascular growth decreases retinal oxygenation and increases vascular signaling, promoting leakage and dysregulated proliferation of immature retinal vessels, which can result in vitreoretinal traction and retinal detachment (135).

Current treatment options for ROP include laser photocoagulation, VEGF inhibitors, and, in severe cases, scleral buckling and/or vitrectomy. All of these carry risks of vision-threatening complications. Less invasive and more effective therapies for ROP are needed. In an oxygen-induced retinopathy model, MSC-EV treatment preserved retinal blood flow, attenuated neovascularization, reduced retinal thinning, and exhibited good tolerability without requiring immunosuppression (136). Intravitreal injection of MSC-EVs alleviates neuroinflammation and cell apoptosis induced by retinal ischemia-reperfusion injury. MSC-EV proteomic analysis detected survival-promoting proteins, such as those involved in the cAMP response element-binding protein pathway (137). Insufficient cAMP response element-binding protein signaling is associated with retinal ischemia and alterations in retinal neurotrophic and inflammatory systems (138). In a preterm retinopathy animal model, EVs derived from microglia can alleviate photoreceptor damage, promoting normal vascular formation, perhaps mediated by miR-24-3p (139). Lymphocyte microparticles attenuate oxygen-induced retinopathy by reducing retinal neovascularization and macrophage infiltration. Lymphocyte microparticle miR-181a may play a regulatory role in retinal vascular neogenesis (140).

## 9 Extracellular vesicles and sepsis

Neonatal sepsis, an invasion of pathogenic microorganisms such as bacteria, triggers a systemic inflammatory response syndrome in the body, leading to potentially severe sequelae and multi-organ damage. Neonatal sepsis is categorized into early-onset sepsis (EOS) and late-onset sepsis based on the time of onset. About 16% of the 2.8 million newborn deaths worldwide are attributed to sepsis (141). Early-onset sepsis accounts for 8% of deaths within the first 7 days of life, while late-onset sepsis is responsible for 37% of deaths occurring after 7 days (141).

EVs feature prominently in sepsis. Bacteria, the primary infectious agents, release bacterial outer membrane vesicles carrying endotoxins into septic patients’ circulatory systems, exacerbating inflammatory responses (142). Increased quantities of host-derived EVs upon bacterial stimulation correlate with sepsis severity (143). In septic mouse serum, EVs encapsulate numerous cytokines and chemokines, and EV inhibitors reduce EV formation and inflammatory cytokine release (144). EVs contribute to multi-organ damage in sepsis, with miR-1262 from septic patients’ EVs inhibiting glycolysis and promoting cardiomyocyte apoptosis (145). Acute lung injury and acute respiratory distress syndrome have upregulated bronchoalveolar lavage fluid and circulating EVs (146). LPS injection in mice increases pulmonary alveolar macrophage EV release, activating NLRP3 inflammasomes and exacerbating sepsis-induced inflammation (147). After LPS stimulation, choroid plexus epithelial cells secrete EVs containing inflammatory proteins and miRNAs, which effect the central nervous system (148).

Diagnosis of early neonatal sepsis requires sensitive and specific biomarkers due to its atypical clinical presentation. Plasma EV levels correlate with organ failure severity and patient outcomes (149). In sepsis, EV-miRNA expression correlates with risk, severity, and prognosis (150). In septic patients’ serum, upregulated circRNA-104484 and circRNA-104670 EVs have diagnostic potential (151). Elevated miRNA-34a and decreased miR-15 and miR-27a in EVs predicts septic shock occurrence (152).

Inhibiting EV generation reduces inflammation and improves the prognosis of septic patient survival (144). Modifying miRNAs in cell derived EVs can modulate the sepsis cytokine storm (153). MSC-EVs carrying anti-inflammatory miRNAs such as miR-17 mitigate LPS-induced inflammation and apoptosis (154). miR30b-3p in MSC-EVs inhibits LPS-induced pulmonary inflammation and enhances cell proliferation (155). MSC-EVs alleviate systemic inflammatory response, improve mouse survival, and protect lung tissues in septic mice (156). LPS-activated macrophages engulf adipose-derived MSC exosomes, inhibiting IL-27 secretion (157).

## 10 Limitations/challenges

To enable the increased use of EVs for their widespread clinical application in the diagnosis and treatment of diseases in premature infants, some key areas that warrant further research include the following:

### 10.1 Isolation methods

The complexity of sample physicochemical properties presents significant challenges for the isolation of EVs (158). Current methods for EV isolation include centrifugation, ultrafiltration, chromatography, immunoseparation, and some commercial kits (159). Alternative isolation methods for EVs have limitations and may affect EV purity and biological activity. For example, the most used differential centrifugation method may not effectively purify EVs from viscous fluids (160), and high-speed centrifugation may lead to co-precipitation of EVs with protein aggregates and apoptotic bodies, resulting in decreased EV purity (161). Ultrafiltration may cause a decrease in EVs yield due to entrapment of exosomes in the pores of the filter membrane, and the force applied to the sample passing through the filter membrane may damage, deform, and rupture large vesicles (162). Immunoseparation is expensive, and it is generally used for isolating cell-free samples because cells or tissues may express similar exosomal membrane markers (163). Therefore, understanding the influence of different isolation methods on the biological activity of EVs is crucial. Developing a unified, efficient, and low-cost method for purifying and scaling up EVs from various samples is crucial.

### 10.2 Therapeutic dose

The therapeutic effect of EVs is dose dependent (164), so quantification of EVs is needed to accurately assess the side effects and therapeutic effects of EV administration. Current quantitative methods include concentrations of reporter proteins, dynamic light scattering, tunable resistive pulse sensing, and nanoparticle tracking analysis; each of these has its advantages and limitations (21). There is currently a lack of uniformity in the quantification of EVs because different researchers often use different parameters to calculate EV doses. Furthermore, subtle variations in tissue culture conditions not only affect the quantity of EVs but also their composition. EVs may be confused with fragments, aggregates, and contaminants, leading to difficulties in quantification. Therefore, rigorous and effective analysis of pre-isolation EVs is needed for accurate quantification (165).

### 10.3 Route of administration

The distribution pattern of EVs in the body depends on the route of administration. Relative to intravenous injection, intraperitoneal and subcutaneous injections have less accumulation of EVs in the liver and spleen but more accumulation in the pancreas and gastrointestinal tract (166). Therefore, defining the optimal administration route for different diseases in premature infants would maximize therapeutic efficacy.

### 10.4 Effects of stimuli on EV contents

Different sources of EVs and differences in the stimuli experienced by a cell type may cause major differences in EV contents, affecting their diagnostic and therapeutic value. For example, preconditioning rat bone marrow MSC under high oxygen conditions *in vitro* has stronger therapeutic effects on lung injury than untreated MSCs (167). Therefore, it is necessary to further compare the therapeutic differences of EVs from different sources and the changes in the contents of EVs under different treatment conditions of the same cells, as well as their effects on the therapeutic efficacy of diseases in premature infants.

### 10.5 Long-term safety and toxicity

Assessing the long-term effects of EVs on immunocompromised premature infants is essential. Large-sample cohort studies and randomized controlled trials are needed to evaluate the long-term effects of EVs on immune function and neurological development.

By addressing these and other unresolved issues, we can maximize our ability to use EVs toward improving the health outcomes of premature infants.

## 11 Future directions

As the field of EVs in preterm-related diseases grows, key areas of research are essential to bridge the gap between laboratory findings and clinical applications. Developing efficient, reproducible, and cost-effective methods for isolating and characterizing EVs is critical (168). Current techniques often compromise purity or yield, requiring advancements in high-throughput technologies to address these challenges (161). Disease-specific EV-derived molecules hold promise for early and non-invasive diagnosis. However, it is not uncommon for specific biomarkers identified in the preclinical phase to fail miserably during clinical validation. Large-scale prospective or multi-cohort studies are warranted to validate diagnostic EV performance and to determine the context of use before integrating it into routine diagnostic workflows (169). For therapeutic purposes, better understanding the molecular mechanisms of selective cargo sorting for miRNAs and proteins is essential for designing tailored EV therapies and optimizing their composition for enhanced functionality (13). Moreover, research should focus on combining EVs with emerging technologies to enhance cargo loading, tissue targeting, and stability, while evaluating their safety, immunogenicity, and long-term effects in preterm infants (170).

## 12 Conclusion

With the recent profound advances in neonatal medicine, including improved survival of very low birthweight premature infants, our Neonatal Intensive Care Unit populations have expanded dramatically. The consequent related morbidity has surged annually, emerging as the leading cause of child mortality and impacting long-term prognoses. EVs are pivotal in intercellular signal transduction, which is a key component in early development. Thus, EVs exert crucial regulatory roles in the pathophysiological processes of spontaneous preterm birth and associated conditions in premature infants. Their unique biological characteristics render EVs promising in disease diagnosis and treatment. However, their widespread clinical application is limited by the current dearth of information regarding composition of various EVs, scaled up EV production, the dose-response relationship of EVs, specifics of treatment modalities, and safety and efficacy of EVs and their components. In the future, we will focus on optimizing EV isolation and characterization techniques, uncovering their biogenesis and cargo sorting mechanisms, developing EV-based non-invasive diagnostic biomarkers, and advancing their therapeutic applications in preterm-related diseases.
